# HPV Genotyping 9G Membrane Test

**DOI:** 10.3390/v5112840

**Published:** 2013-11-22

**Authors:** Danishmalik Rafiq Sayyed, Keum-Soo Song, Satish Balasaheb Nimse, Heejung An, Junghoon Kim, Taisun Kim

**Affiliations:** 1 Institute for Applied Chemistry and Department of Chemistry, Hallym University, Chuncheon, 200-702, Korea; E-Mail: tskim@hallym.ac.kr; 2 Biometrix Technology, Inc. 202 BioVenture Plaza, Chuncheon, 200-161, Korea; E-Mail: hanlimsk@empal.com; Fax: +82-33-256-3421; 3 Department of Pathology, College of Medicine, CHA University, 487-801, Seongnam, Korea; E-Mail: hjhan@cha.ac.kr

**Keywords:** HPV, cervical neoplasia, genotyping, 9G membrane, cervical cancer

## Abstract

The results of the genital human papillomavirus (HPV) detection in 439 cervical samples by cervical cytology were compared with sequencing analysis and a newly developed HPV genotyping 9G membrane test. The excellent sensitivity and specificity of the HPV genotyping 9G membrane test was assured by a signal to noise ratio of more than 300 and a target hybridization to non-target hybridization ratio of 300 ~ 400 at 25 °C. The final results can be obtained in 29 min by simple loading of the hybridization and washing solutions and scanning the membranes without any drying steps or special handling. The 100% identical results of the HPV genotyping 9G membrane test with sequencing results in 439 clinical samples demonstrate significant clinical application for this test. HPV genotyping 9G membrane tests can identify and discriminate five HR-HPV genotypes which are prevalent in almost 87% of cervical cancer cases. Its simple handling makes the HPV genotyping 9G membrane test a very convenient platform for accurate HPV genotyping.

## 1. Introduction

Genital human papillomavirus (HPV) is the most common sexually transmitted infection in the United States, with an estimated 6.2 million people becoming newly infected every year [1]. Cervical cancer caused by HPV infection is the second most common cancer in terms of both incidence and mortality worldwide [2]. Estimated new cases and deaths from cervical cancer due to HPV infections in the United States in 2000 are 12,800 and 4500, respectively, which indicates a mortality rate of 35% [3,4]. Molecular epidemiologic evidence clearly indicates that certain types of high-risk human papillomavirus (HR-HPV) are the principal cause of the invasive cervical cancer [5] and the cervical intraepithelial neoplasia [6,7]. Eighty-seven percent of cervical cancer cases were detected to be infected with HPV16, HPV18, HPV45, HPV31, and HPV33 [8]. Therefore, a highly proficient HPV test that can genotype the high-risk HPV is essential. 

However, traditional methods for HPV detection, such as morphological and immunological methods, lack the ability to detect specific HPV types [9]. Real-time PCR based methods are widely used, however the clinical sensitivity and specificity of these methods is very low and hence unsuitable for early clinical diagnosis of HPV infection [10]. Sequencing analysis is a “gold standard” for the detection of genotypes in clinical samples. However, the major disadvantage of these methods is that they need expensive instrumentation and highly trained professionals. 

The high mortality rate in HPV infected patients demands the urgency to develop a rapid and field-deployable HPV genotyping test [11]. Ideally, to be of general field utility, HPV genotyping test must be capable of sensitive and specific HPV detection while retaining simplicity of use and independence from complex laboratory instrumentation.

Recently, we have reported the 9G DNAChip technology [12] for the genotyping of highly pathogenic viruses such as human papillomavirus (HPV) [13,14] and human influenza virus (H1N1) [15]. Though this technology is highly reproducible and allows the HPV 9G DNAChips to get 100% clinical sensitive and specific genotyping results in clinical samples [16], the use of costly instrumentation and highly trained professionals limits its use in laboratory settings. Therefore, to address these problems a HPV genotyping 9G membrane test was developed for simple and convenient HPV genotyping. Final results can be obtained directly by loading the hybridization and washing solutions. 

The HPV genotyping 9G membranes for the rapid detection and discrimination of the HPV genotypes were tested in 439 clinical samples. It is for the first time that the genotyping results are obtained by the direct hybridization of the Cy5 labeled PCR products to the immobilized probes on the membrane. The HPV genotyping 9G membranes show high sensitivity, 100% target-specific hybridization, discrimination of the genotypes with a ratio of 360:1, at 25 °C in 28 min, and does not need a complex and expensive instrumentation. The HPV genotyping 9G membrane test can effectively detect the five HR-HPV genotypes which are prevalent in cervical cancer cases. The HPV genotyping 9G membrane is basically designed for the detection and discrimination of the five HR-HPV genotypes. Moreover, the HPV2 genotyping 9G membrane can screen the presence or absence of the other 14 HR-HPV genotypes.

## 2. Results and Discussion

### 2.1. HPV Detection and Genotyping by the Sequencing

**Figure 1:**
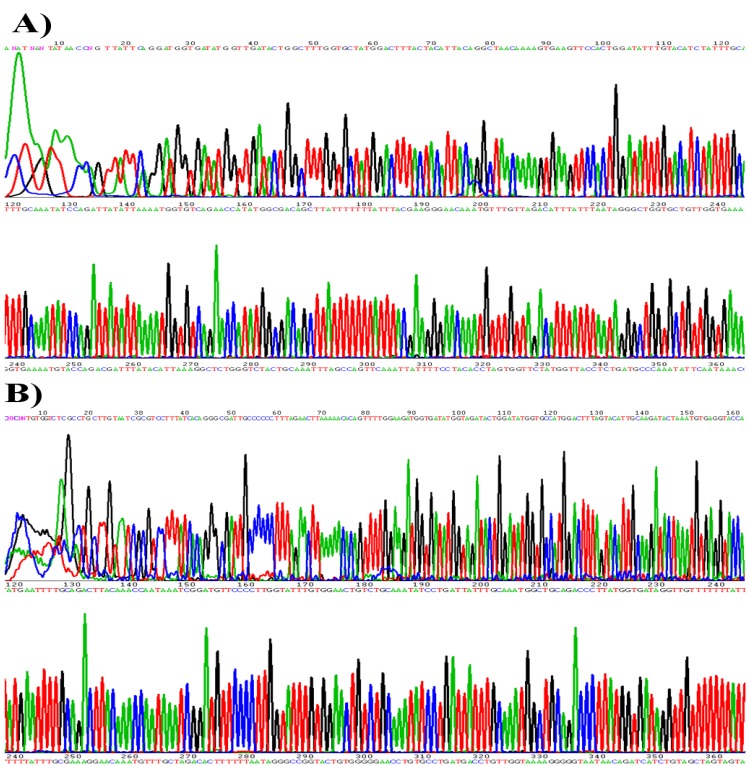
Human papillomavirus (HPV) genotyping by the sequencing analysis **(a)** HPV-16; **(b)** HPV-18.

The primed PCR product was added to the sequencing reaction mixture. Sequencing was performed bidirectionally with the BigDye3 terminator cycle sequencing kit (PE Applied Biosystems) using ABI PRISM 310 Genomic Analyser (PE Applied Biosystems) at a dispensing pressure of 600 mbar with 8-ms open times and 65-s cycle times. The sequencing procedure was carried out by stepwise elongation of the primer strand upon cyclic dispensation of the different deoxynucleoside triphosphates (Amersham Pharmacia Biotech). A CCD camera detected the light output resulting from nucleotide incorporation. The data were obtained in a graphic format (Figure 1). Out of the 439 clinical samples 140 samples were found to be HPV positive in the sequencing analysis.

### 2.2. HPV Genotyping 9G Membrane Test and HPV2 Genotyping 9G Membrane Test

The HPV genotyping 9G membranes consist of the five HR-HPV type specific probes (HPV-16, HPV-18, HPV-45, HPV-31, and HPV-33). Whereas, the HPV2 genotyping 9G membrane consist of the 14 HR-HPV type specific probes (Line A: HPV-66, HPV-52; Line B: HPV-59, HPV-56; Line C: HPV-35, HPV-51, HPV-58, HPV-68, HPV-69; Line D: HPV-70, HPV-39; and Line E: HPV-73, HPV-53, HPV-26). The 5 µl of PCR product was subjected to agarose gel electrophoresis, using a 2% agarose gel, the product size of HPV DNA was found to be 250 base pairs (bp). 

**Scheme 1:**
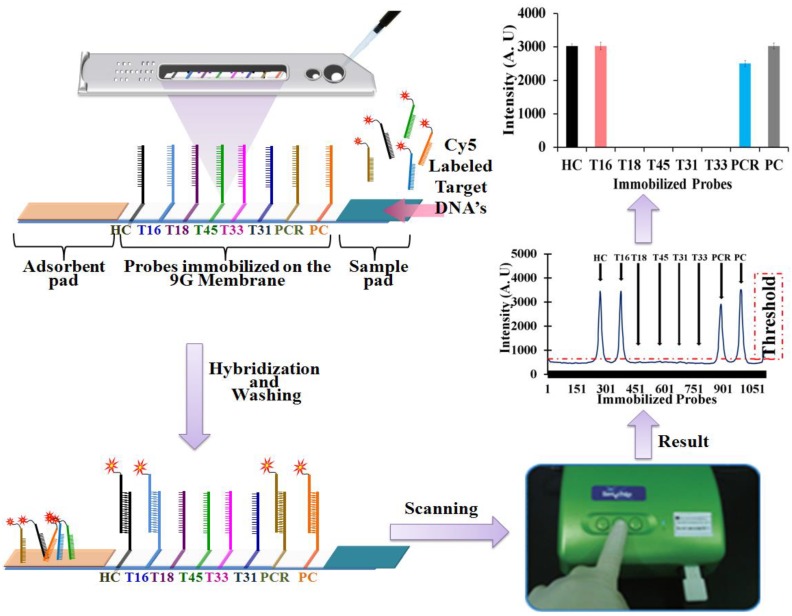
HPV genotyping 9G membrane, hybridization, washing, scanning, and result.

**Scheme 2:**
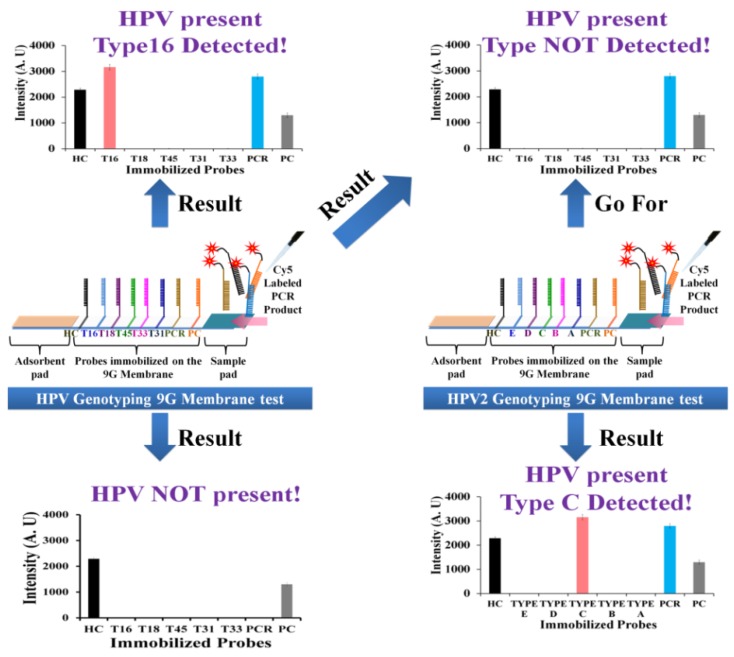
Detection and discrimination of the HR-HPV genotypes by HPV genotyping 9G membrane and HPV2 genotyping 9G membrane.

For the detection and discrimination of the HPV genotypes in the clinical samples the 110 μL of hybridization mixture containing Cy5 labeled PCR product of HPV genotype (e.g., HPV16, HPV18 *etc.*) was loaded into the sample loading port on the HPV genotyping 9G membrane strip. If necessary another 110 μL of hybridization mixture containing Cy5 labeled PCR product was loaded on the HPV2 genotyping 9G membrane strips. The hybridization, washing and scanning procedure was followed as mentioned earlier. Each experiment was done more than three times. HPV amplicons can be hybridized with type-specific oligonucleotide probes on HPV genotyping 9G membrane and can visualized on the graph after analysis of results (Scheme 1, Scheme 2).

The HPV genotyping 9G membrane can detect five HR-HPV genotypes. After hybridization of the PCR products with probes immobilized on the HPV genotyping 9G membrane, the three possible results as shown in the Scheme 2 can be observed. (i) HPV present, specific type detected: the results of scanning can demonstrate the fluorescence intensities for the HC, PC, PCR, and a specific HR-HPV type (e.g., HPV16, HPV18, HPV45, HPV31, and HPV33). (ii) HPV NOT present: the results of scanning can demonstrate the fluorescence intensities only for the HC, PC. (iii) HPV present Type NOT detected: the results of scanning can demonstrate the fluorescence intensities for the HC, PC, and PCR.

**Table 1:** Comparison of the results of HPV genotyping by the Cervical Cytology with the results of the sequencing analysis, HPV genotyping 9G membrane test (439 clinical samples).

Cervical Cytology		Sequencing		HPV genotyping 9G membrane test	
		HPV +	HPV -	HPV +	HPV -
**Normal**	362	74 (20.4%)	288	74 (20.8%)	288
**ASC-US**	47	39 (83.0%)	8	39 (83.0%)	8
**ASC-H**	9	7 (77.8%)	2	7 (77.8%)	2
**LSIL**	9	8 (88.9%)	1	8 (88.9%)	1
**HSIL**	12	12 (100%)		12 (100%)	

Note: ASC-US—atypical squamous cells of undetermined significance; ASC-US-H—atypical squamous cells cannot exclude HSIL; LSIL—low-grade squamous intraepithelial lesion; HSIL—high-grade squamous intraepithelial lesions.

**Table 2:** Comparison of the results of HPV genotyping by the sequencing analysis and HPV genotyping 9G membrane test in the 439 clinical samples.

HPV genotyping 9G membrane Test		Normal		ASC-US		ASC-H		LSIL		HSIL	
		A	B	A	B	A	B	A	B	A	B
	**HPV Type**										
	HPV 16	11	11	7	7	3	3	2	2	7	7
	HPV 18	4	4	2	2						
	HPV 45	2	2								
	HPV 31	2	2	2	2			1	1	1	1
	HPV 33	2	2	1	1	1	1	1	1		
**HPV2 genotyping 9G membrane Test**											
**Line A**	HPV 66	2	2	2	2						
	HPV 52	11	11	7	7	2	2				
**Line B**	HPV 59										
	HPV 56	2	2	4	4			2	2		
**Line C**	HPV 35			1	1						
	HPV 51	1	1	1	1			1	1		
	HPV 58	5	5	6	6	1	1			4	4
	HPV 68	5	5	1	1						
	HPV 69										
**Line D**	HPV 70										
	HPV 39	7	7	1	1			1	1		
**Line E**	HPV 73										
	HPV 53										
	HPV 26										
**NOT HR-HPV Types**		20	20	4	4						
**Negative**		288	288	8	8	2	2	1	1		

Note: A—Results of sequencing analysis; B—Results of HPV genotyping 9G membrane test; ASC-US—atypical squamous cells of undetermined significance; ASC-US-H—atypical squamous cells cannot exclude HSIL; LSIL—low-grade squamous intraepithelial lesion; HSIL—high-grade squamous intraepithelial lesions.

In the case of the (i) and (ii) results, the use of the HPV2 genotyping 9G membrane test is not necessary as the HPV genotyping 9G membrane test can either detect the absence of HPV genotype or presence of at least one HR-HPV genotype out of five HR-HPV genotypes. However, in the case of result (iii), the hybridization mixture containing same PCR product should be loaded on the HPV2 genotyping 9G membrane test for further detection of the HR-HPV genotype out of 14 HR-HPV genotypes.

**Figure 2:**
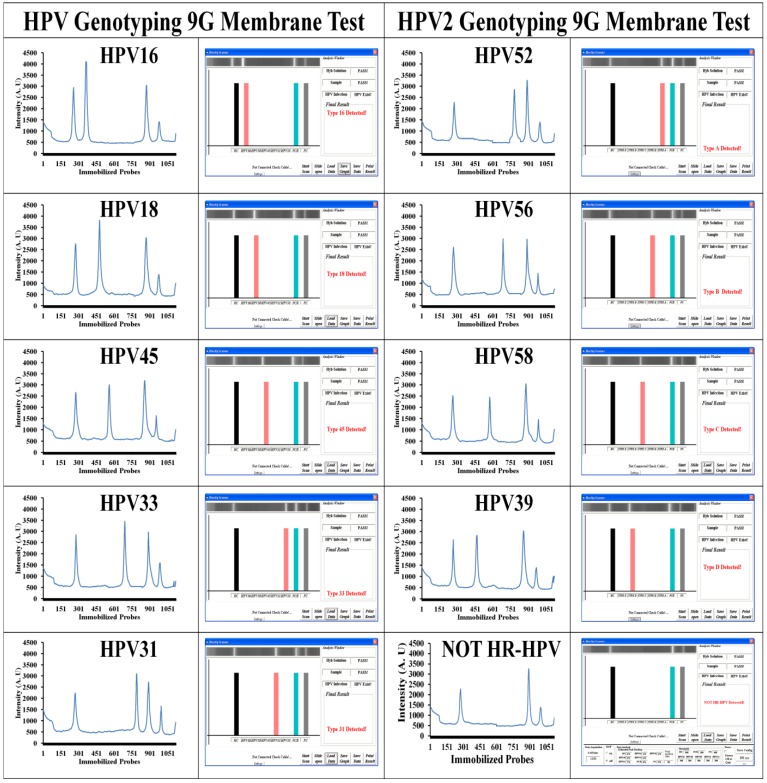
HPV genotyping by the HPV genotyping 9G membrane. Respective graphs of fluorescence intensities after the hybridization of the immobilized probes on HPV genotyping 9G membrane with the Cy5 labeled PCR products of the HPV genotypes.

In the case of the HPV2 genotyping 9G membrane test, two possible results as shown in the Scheme 2 can be observed. (i) HPV present Type C (e.g., A, B, D, or E) detected: the results of scanning can demonstrate the fluorescence intensities for the HC, PC, PCR and Type C. (ii) HPV present Type NOT detected: the results of scanning can demonstrate the fluorescence intensities for the HC, PC, and PCR. In the case of result (ii), the presence of the fluorescence intensity for PCR indicate that the clinical sample does contain the HPV genotype except for the 19 HR-HPV genotypes which can be detected by both types of membranes. Therefore, in such cases, the presence of the HPV genotype in the clinical sample is recorded as NOT HR-HPV genotype in the final results as shown in Table 2. The results of the HPV 9G membrane tests and the HPV2 9G membrane tests are presented in Figure 2 and Table 1 and Table 2. 

Interestingly, the excellent specificity of the probes immobilized on the HPV genotyping 9G membranes ensured a SBR of more than 300 (Figure 2) which is superior to the reported HPV detection tools. The target hybridization provides 300 ~ 400 times stronger signal intensity as compared to the non-target hybridization. The HPV genotyping 9G membranes allowed the genotyping of a number of HPVs in one reaction. This is advantageous for the diagnostic purposes when genotyping HPV in clinical samples.

**Figure 3:**
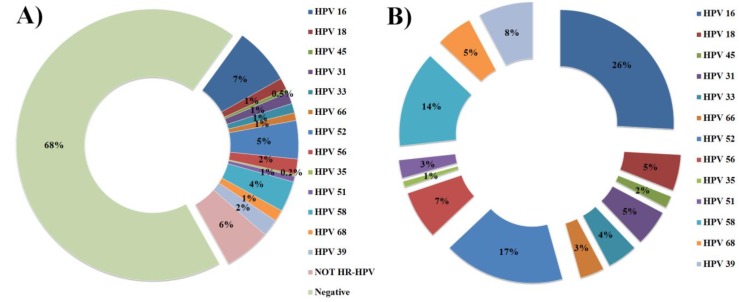
Results of sequencing analysis and the HPV genotyping 9G membrane test. **(a)** Distribution of HPV genotypes in 439 clinical samples; **(b)** Distribution of the HPV genotypes in 140 HR-HPV positive clinical samples.

The results of the HPV genotyping by the HPV genotyping 9G membrane tests were compared with the results of the HPV genotyping by the sequencing analysis and the cervical cytology in the 439 clinical samples, in which specific HPV genotypes were, detected (Table 1). Out of the 439 clinical samples, 140 samples (32%) were found to be HPV positive in the sequencing analysis. These results were 100% identical with that of the HPV genotyping 9G membrane tests (Table 1 and Table 2).

Out of 140 HPV positive cases, 83% were detected to be HR-HPV genotypes and the 17% were detected to be NOT HR-HPV genotypes. The distribution of the HPV genotypes in the 439 clinical samples is demonstrated in Figure 3. The results of the sequencing test for the HPV genotypes such as HPV-16 (26%), HPV-52 (17%), HPV-58 (14%), HPV-39 (8%), HPV-56 (7%), HPV-18 (5%), and HPV-31 (5%) were 100% identical with the HPV genotyping 9G membrane test.

**Figure 4:**
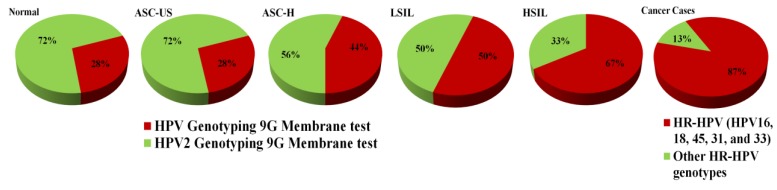
HPV genotypes and their clinical significance in ASC-US, ASC-US-H, LSIL, and cancer cases.

The comprehensive analysis of the results presented in Figure 4 and Table 1 provides important information which has a high clinical significance in detection of the HR-HPV genotypes in the clinical samples. Figure 4 demonstrates the HPV genotypes and their clinical significance in ASC-US, ASC-H, LSIL, HSIL, and cancer cases. It is well known that almost 87% cases of cervical cancers are infected with the five HR-HPV types (HPV16, 18, 45, 31, and 33) and 13% of the cervical cancers are infected with other HPV types (Figure 4, cancer cases). From the results of the present study, Figure 4 also shows that the percentage of the HR-HPV types (HPV16, 18, 45, 31, and 33) is 28% in the ASC-US (atypical squamous cells of undetermined significance) which is an early stage of HPV infection and increases through ASC-H (44%), LSIL (50%) to HSIL (67%) (high-grade squamous intraepithelial lesions). It is very important to note that the HPV genotyping 9G membrane tests play a vital role in the detection and discrimination of the important HR-HPV genotypes which are the major infectious agents found in cervical cancer cases. The pattern of the increase in the percentage of the HPV16, HPV18, HPV45, HPV31, and HPV33 from ASC-US to HSIL demonstrates the importance of HPV genotyping 9G membrane tests. Therefore, the HPV genotyping membrane plays a vital role in the clinical diagnosis of HPV infection as it can detect and discriminate the five most important HR-HPV genotypes. The HPV2 genotyping 9G membrane tests can detect the remaining HR-HPV genotypes, other than the five major HR-HPV genotypes.

### 2.3. Statistical Analysis

The accuracy of the HPV genotyping 9G membrane tests for the detection of the HPV genotypes in 439 clinical samples was calculated from the sensitivity (true positive rate) and specificity (true negative rate) of 95% confidence intervals (CI). Considering the fact that HPV genotyping 9G membrane tests showed 100% identical results with the sequencing analysis, the samples that were positive by sequencing analysis and the HPV genotyping 9G membrane tests were defined as true positive and specimens negative by both of these methods were defined as true negative. The sensitivity and specificity were calculated by assuming that the sequencing analysis of the clinical samples was correct.

### 2.4. Discussion

Interestingly, the excellent specificity of the probes immobilized on the HPV genotyping 9G membranes ensured the SBR of more than 300 (Figure 2) which is superior to the reported HPV detection tools. The target hybridization provides 300 ~ 400 times stronger signal intensity as compared to the non-target hybridization, which serves as a key for the proficient genotyping test by HPV genotyping 9G membranes.

The 100% clinical sensitivity as well as 100% clinical specificity was observed for the HPV genotyping by the HPV genotyping 9G membrane tests. The clinical sensitivity and specificity of the cervical cytology was 67% and 80%, respectively. It is evident from the results of HPV genotyping 9G membrane test that many clinical samples designated as normal in cervical cytology were found to be high risk type HPV genotypes (20%). The low sensitivity and specificity of cervical cytology may lead to improper diagnosis. On the contrary, the HPV genotyping 9G membrane test shows profound accuracy in genotyping HPVs in clinical samples.

The major disadvantage of commercial DNA chip based HPV genotyping tests is that they require a complicated washing and drying step which requires highly trained professionals. However, in the case of HPV genotyping 9G membrane tests, hybridization solution and washing solution are directly loaded on the membranes. The final results can be obtained by scanning the membranes without any drying steps or special handling. These simple handling steps make HPV genotyping 9G membrane tests a very convenient platform for accurate HPV genotyping.

The 100% sensitivity and specificity in the detection and discrimination of the HR-HPV genotypes establish the accuracy of HPV genotyping 9G membrane tests in the clinical diagnosis of HPV infections. The following factors probably contribute to proficient genotyping by the presented test: (i) hybridization, washing, and direct scanning at 25 °C, (ii) high SBR of 300 as compared to those of 2.5–5 for the other tests, (iii) the 100% target-specificity in terms of the 100% identical results with sequencing analysis.

The results for sequencing of the HPV genotypes HPV-16 (26%), HPV-52 (17%), HPV-58 (14%), HPV-39 (8%), HPV-56 (7%), HPV-18 (5%), and HPV-31 (5%) were 100% identical with the HPV genotyping 9G membrane test. The 67% (HPV16 58.3%, HPV318.7%) of HR-HPV genotypes which were found to cause HSIL were detected by the HPV genotyping 9G membrane test and the remaining 13% (HPV58) was detected by the HPV2 genotyping 9G membrane test. Similarly, the 60% (HPV16 30%, HPV3 15%, and HPV33 15%) of HR-HPV genotypes which were found to cause LSIL were detected by the HPV genotyping 9G membrane test and the remaining 40% (HPV56 20%, HPV51 10%, and HPV39 10%) were detected by the HPV2 genotyping 9G membrane test. 

It is interesting to note that the results of cervical cytology demonstrates that, out of 439 samples, 362 were normal and others were ASC-US (47 samples), ASC-H (9 samples), LSIL (9 samples), and HSIL (12 samples) (Table 2). However, the HPV genotyping 9G membrane test demonstrates that 74 samples out of the 362 samples found normal in cervical cytology were actually infected with the HPV genotypes. The ability of HPV genotyping 9G membrane tests to detect the HPV genotypes in the clinical samples is attributed to its limit of detection. The limit of detection of the HPV genotyping 9G membrane test for the detection of four HPV genotypes (HPV16, HPV18, HPV31, and HPV33) is 10^1^ copies and other HPV genotypes including HPV45 is 10^2^ copies [17]. 

HPV genotyping tests based on the HPV genotyping 9G membrane tests are accurate methods for detection and genotyping of HPV. The genotypes of HPV genotyping 9G membrane tests were in 100% agreement with genotypes of HPV DNA sequencing. The HPV genotyping 9G membrane tests have proved that the preliminary findings of the cervical cytology of HPV infected samples may lead to the false detection of HPV. Out of 362 clinical samples, which were designated as normal (HPV negative) by cervical cytology, the 72 samples (20.4%) were identified as HPV positive in the HPV genotyping 9G membrane tests.

The TaqMan PCR or other PCR techniques also represent a sensitive method for the detection of HPV DNA. Currently, standard nested HPV PCR can be performed using degenerative primers followed by direct sequencing of the PCR product. Alternatively, PCR products can be hybridized to DNA of known HPV types, to determine the type of HPV present in the PCR amplified sample (e.g., Roche HPV Amplicor system). Both assays are time consuming and often associated with false positive results.

The present HPV genotyping 9G membrane test is designed for the detection and discrimination of the five HR-HPV genotypes. However, it can effectively identify and discriminate five HR-HPV (HPV16, HPV18, HPV45, HPV31, and HPV33) genotypes which are prevalent in almost 87% of cervical cancer cases. Moreover, the HPV2 genotyping 9G membrane can screen the presence or absence of the other 14 HR-HPV genotypes in clinical samples.

However, the HPV genotyping 9G membrane tests take only 29 min for highly specific detection of HPV genotypes. Moreover, the advantage of HPV genotyping 9G membrane test is that it uses a simple hybridization and washing step which allows it to be used in small laboratories, and does not need highly trained professionals.

## 3. Experimental Section

### 3.1. Materials

Glass fiber membrane (2.5 × 7.5 cm) was purchased from Whatman, Springfield, UK**.** All oligonucleotides were purchased from Bioneer, Korea. All chemicals were purchased from Sigma-Aldrich Chemicals, Korea. All washing solvents for the substrates are of HPLC grade from SK Chemicals, Korea. Ultrapure water (18 M Ω/cm) was obtained from a Milli-Q purification system (Millipore, Billerica, MA, USA).

### 3.2. Instruments

Oligonucleotides were lined using dispenser (BioDot Technologies, Inc., 2852 Alton Pkwy, Irvine, CA 92606, USA). Hybridization was done at 25 °C and no special instrument is required. The fluorescence signal intensities were measured and analyzed by BMT Membrane Reader^TM^ (Biometrix Technology Inc., Korea).

### 3.3. Composition of Different Solutions Used

(a) Immobilization solution (pH = 7.4): 15% glycerol, 50 mM butyl amine, 600 mM NH_4_Cl, (b) Blocking buffer solution (pH = 7.4): 0.5% milk casein in 4× SSC, (c) Hybridization buffers (pH = 7.4): 25% Formamide, 0.1% Triton X-100, 6x SSC, d) Washing buffer solution (pH = 7.4): 0.1% SDS in 4× SSC.

### 3.4. Typical Method for Preparation of the HPV Genotyping 9G Membranes

The HPV genotyping 9G membranes were obtained by slight modification in the 9G DNAChip technology used for the fabrication of the 9G DNAChips. Unlike the nitrocellulose membranes [18] in other methods, a glass membrane was used to produce 9G membrane because the glass membranes are similar to the slide glass in the physicochemical properties. Therefore, the immobilization mechanism of oligonucleotide probes on the DNA chips can be reproduced on the glass membranes. By lining the 18 pmol/µL solution of the oligonucleotide Probe1–Probe8 appended with nine consecutive guanines (Table 3) the oligonucleotides can be immobilized on the AMCA membrane in 4 h. 

**Table 3:** Sequences of the probes immobilized on the HPV genotyping 9G membranes

HPV genotyping 9G membrane			
Line	Probes	Type	Sequence
**T16**	**Probe 1**	HPV16	5’-GGGGGGGGG TTTTTTTTT GTA CCT ACG ACA AGG GGA GG-3’
**T18**	**Probe 2**	HPV18	5’-GGGGGGGGG TTTTTTTTT GTA TAG CAG ACT TGT TGA GG-3’
**T45**	**Probe 3**	HPV45	5’-GGGGGGGGG TTTTTTTTT GTA TAG TAG ACA AGT GGA GG-3’
**T33**	**Probe 4**	HPV33	5’-GGGGGGGGG TTTTTTTTT ATA TAT AAG ACA AGT TGA AG-3’
**T31**	**Probe 5**	HPV31	5’-GGGGGGGGG TTTTTTTTT GTA TTT AAG ACA AGG TGA GG-3’
**HC**	**Probe 6**	HC	5’-GGGGGGGGG CTTTATTTT CC ACT GTT CTC GGC ACG-3’
**PCR**	**Probe 7**	PCR	5’-GGGGGGGGG CTTTATCTT GAC ATG KKG ARG ART ATG A-3’
**PC**	**Probe 8**	PC	5’-GGGGGGGGG TGATTT ACA GTT TAT DTT TC-3’
**HPV2 genotyping 9G membrane**			
**Line**	**Probes**	**Type**	**Sequence**
**Line-A**	**Probe 9**	HPV66	5’-GGGGGGGGG TTTTTTTTT ATA CCT TCG CCA AGT GGA GG-3’
	**Probe 10**	HPV52	5’-GGGGGGGGG TTTTTTTTT ATA CCT TCG TCA TGG CGA GG-3’
**Line-B**	**Probe 11**	HPV59	5’-GGGGGGGGG TTTTTTTTT ATA TGC CAG ACA AGT GGA GG-3’
	**Probe 12**	HPV56	5’-GGGGGGGGG TTTTTTTTT GTA CCT TAG ACA AGT GGA GG-3’
**Line-C**	**Probe 13**	HPV35	5’-GGGGGGGGG TTTTTTTTT ATA TTT AAG GCT TGG TGA AG-3’
	**Probe 14**	HPV51	5’-GGGGGGGGG TTTTTTTTT ATA TAT TAG GCA TGG GGA AG-3’
	**Probe 15**	HPV58	5’-GGGGGGGGG TTTTTTTTT ATA TGT ACG TCA TGT TGA AG-3’
	**Probe 16**	HPV68	5’-GGGGGGGGG TTTTTTTTT ATA TAT TAG GCA TGT TGA GG-3’
	**Probe 17**	HPV69	5’-GGGGGGGGG TTTTTTTTT GTT TAT AAG GCA TGG TGA GG-3
**Line-D**	**Probe 18**	HPV70	5’-GGGGGGGGG TTTTTTTTT ATA TAC TAG GCT TGT GGA GG-3’
	**Probe19**	HPV39	5’-GGGGGGGGG TTTTTTTTT ATA TAC CAG GCA CGT GGA GG-3’
**Line-E**	**Probe 20**	HPV73	5’-GGGGGGGGG TTTTTTTTT ATA TTT AAG ACA AGC AGA AG-3’
	**Probe 21**	HPV53	5’-GGGGGGGGG TTTTTTTTT GTA TGT TAG ACT TGC AGA GG-3’
	**Probe 22**	HPV26	5’-GGGGGGGGG TTTTTTTTT ATT TAT AAG ACA TGG CGA AG-3’
**Target1 ( HC-Cy5-T1)**		HC-Cy5	3’-GGATCACCGAGATACCATTGGAGACTGCG-Cy5-5’
**Forward primer**		FP	3`-GCMCAGGGWCATAAYAATGG-5’
**Reverse primer**		RP-Cy5	3`-GAAAHATAAACTGTAAATCATAYTC-Cy5-5’

Note: HC—probe for the Hybridization control; PC—probe for the Primer control (Positive control); PCR—probe for the PCR control; HC-Cy5-T1—Target oligonucleotide for HC probe; GGGGGGGGG—9G for immobilization of the probes on the AMCA slides; CTT TAT TTT, TTTTTTTTT—vertical spacer groups

After immobilization, the membranes were soaked in the blocking solution and then dried to generate the HPV genotyping 9G membrane (Scheme 1). Similarly, by lining the Probe 6–8 and Probe 9–22, the HPV2 genotyping 9G membranes were obtained. These membranes were assembled in to the strips. In the case of HPV2 genotyping 9G membranes, the group of probes were mixed and lined on the membrane as follows. On the Line-A line, the mixture of Probe 9 and Probe 10 was lined. On the Line-B line, the mixture of Probe 11 and Probe 12 was lined. On the Line-C line, the mixture of Probe 13–17 was lined. On the Line-D line, the mixture of Probe 18 and Probe 19 was lined. On the Line-E line, the mixture of Probe 20–22 was lined. Besides the probes complementary to the HPV genotypes, the probes (Probe 6–8) corresponding to the hybridization control (HC), PCR control (PCR,) and positive control (PC) were also lined on both membranes.

### 3.5. General Procedure for Hybridization, Washing, and Scanning

The hybridization buffer was prepared by mixing the 20 mL of hybridization solution and 600 μL of Cy5-HC-T1 (60 fmol/μL). A final 240 μL of hybridization mixture was prepared by mixing the 220 μL of the hybridization buffer and 20 μL of Cy5 labeled PCR product of HPV genotype (e.g., HPV16, HPV18). Out of 240 μL, the 110 μL of this hybridization mixture was loaded on sample loading port on the HPV genotyping 9G membrane strip and allowed to hybridize for 20 min at 25 °C. After hybridization, washing solution was loaded into the washing port and allowed to stand for 8 min. Then, the 9G membranes were scanned by BMT Membrane Reader^TM^ to obtain final results. Each experiment was done more than three times. The genotyping results can be obtained in 29 min by using HPV genotyping 9G membrane tests.

### 3.6. Clinical Samples

The samples were collected consecutively from 439 Korean women who visited the Department of Obstetrics and Gynecology, CHA Clinic, South Korea. The samples were collected by scraping the uterine cervical canal with a small cytobrush after Pap smear, and the brush was put into a 15 mL centrifuge tube containing phosphate-buffered saline. The specimens were collected as part of an informed consent protocol approved by the clinical studies committee of the CHA Bundang hospital, South Korea.

### 3.7. Study Subjects

The cervical cytology was performed on all 439 cervical samples. Out of 439 samples, the HPV positive results were found in 77 samples and the remaining 362 samples were found as normal (No HPV detected) (Table 1). The HPV positive 77 samples were categorized as ASC-US (47, 61.0%), ASC-US-H (9, 11.7%), LSIL (9, 11.7%), and HSIL (12, 15.6%). The results of the cervical cytology were compared with the results of the sequencing analysis, and HPV genotyping 9G membrane test results (Table 2).

### 3.8. DNA Extraction and (PCR) Amplification

The MY09/MY11 primer set-mediated PCR (MY-PCR) and the GP5^+^/GP6^+^ primer set-mediated PCR (GP^+^-PCR) are the most frequently used amplification systems for the detection of HPV DNA in clinical samples, amplifying DNA fragments in the conserved L1 region with approximately 450 bp and 150 bp, respectively. Further, type-specific PCR primer sets allow the identification of individual genotypes. The MY11/ GP6^+^ primer set consists of a fixed nucleotide sequence for the forward and reverse primers, respectively, and detects a wide range of HPV types by using a lowered annealing temperature during PCR [19,20].

The whole HPV genomic DNA extracted from clinical samples was amplified by duplex PCR to generate amplicons. HPV DNA was amplified with primers RP and FP (Table 3). The PCR mixture consisted of 10 µL of the extracted DNA, 10 µL of each primer (RP, FP), PCR premix (cat# K-2016V1, Bioneer Inc., Daejan, Korea) containing deoxyribonucleotide triphosphate, 2U of Fast Start *Taq* DNA polymerase in an amplification buffer containing 2mM MgCl_2_, and a tracking dye (Cy5). All tubes were incubated for 2 min at 50 °C before PCR was started. Amplification was performed with the following steps: pre-denaturation for 5 min at 94 °C, 45 cycles of 30 s each for the denaturation at 94 °C; 45 cycles of 30 s each for annealing at 65 °C, 45 cycles of 30 s each for elongation at 72 °C, and an final elongation step of 7 min at 72 °C. 5 µL of PCR product was subjected to agarose gel electrophoresis, using a 2% agarose standard run in 1X Tris borate EDTA. 10 μl of this Cy5 labeled PCR product was used for the further hybridization experiments in the HPV genotyping 9G membrane tests for the HPV detection and genotyping. The whole procedure was followed for each of the 439 clinical samples.

## 4. Conclusions

The excellent sensitivity and the specificity of the HPV genotyping 9G membrane tests were assured by a signal to noise ratio of more than 300 and a target hybridization to non-target hybridization ratio of 300 ~ 400. In the case of HPV genotyping 9G membrane tests, the hybridization and washing solutions are directly loaded on the membranes. The final results can be obtained by scanning the membranes without any drying steps or special handling. These simple handling steps make the HPV genotyping 9G membrane tests a very convenient platform for accurate HPV genotyping.

Furthermore, in this study, the accuracy of HPV genotyping 9G membrane tests for HPV genotyping could be certified by comparison with the sequencing data, which is found to be 100% identical in all cases. The HPV genotyping 9G membrane tests demonstrate a clinical value in decision-making as they can identify and discriminate HR-HPV genotypes which are prevalent in almost 87% of cervical cancer cases. The efficient detection and discrimination of the 439 clinical samples make HPV genotyping 9G membrane tests a promising diagnostic tool for accurate HPV genotyping.
